# Framework for the Development of Data-Driven Mamdani-Type Fuzzy Clinical Decision Support Systems

**DOI:** 10.3390/diagnostics9020052

**Published:** 2019-05-09

**Authors:** Yamid Fabián Hernández-Julio, Martha Janeth Prieto-Guevara, Wilson Nieto-Bernal, Inés Meriño-Fuentes, Alexander Guerrero-Avendaño

**Affiliations:** 1 Facultad de Ciencias Económicas, Administrativas y Contables, Universidad del Sinú Elías Bechara Zainúm, Montería, Córdoba 230001, Colombia; 2 Departamento de Ciencias Acuícolas–Medicina Veterinaria y Zootecnia (CINPIC), Universidad de Córdoba, Montería, Córdoba 230001, Colombia; mprieto@correo.unicordoba.edu.co; 3 Facultad de Ingeniería, Departamento de Ingeniería de Sistemas, Universidad del Norte, Puerto Colombia, Atlántico 080001, Colombia; wnieto@uninorte.edu.co (W.N.-B.); imerino@unimagdalena.edu.co or imerino@uninorte.edu.co (I.M.-F.); 4 Facultad de Ingeniería, Departamento de Ingeniería de Sistemas, Universidad del Magdalena, Santa Marta, Magdalena 470001, Colombia; 5 Facultad de Ingeniería, Universidad Francisco de Paula Santander, Cúcuta, Santander 540001, Colombia; aguerreroav@ufpso.edu.co

**Keywords:** clusters, rule base, knowledge base, fuzzy sets, deep learning

## Abstract

Clinical decision support systems (CDSS) have been designed, implemented, and validated to help clinicians and practitioners for decision-making about diagnosing some diseases. Within the CDSSs, we can find Fuzzy inference systems. For the reasons above, the objective of this study was to design, to implement, and to validate a methodology for developing data-driven Mamdani-type fuzzy clinical decision support systems using clusters and pivot tables. For validating the proposed methodology, we applied our algorithms on five public datasets including Wisconsin, Coimbra breast cancer, wart treatment (Immunotherapy and cryotherapy), and caesarian section, and compared them with other related works (Literature). The results show that the Kappa Statistics and accuracies were close to 1.0% and 100%, respectively for each output variable, which shows better accuracy than some literature results. The proposed framework could be considered as a deep learning technique because it is composed of various processing layers to learn representations of data with multiple levels of abstraction.

## 1. Introduction

Clinical decision support systems (CDSS) have been designed, implemented, and validated to help clinicians and practitioners with decision-making about diagnosing some diseases by managing data or medical knowledge [1]. Within these CDS systems, we can find several techniques: Machine Learning (ML) [2], Deep Learning (DL) [3], and Fuzzy logic (FL) systems [4,5]. According to Reference [2], among the first techniques, we can find k-Nearest Neighbor—k-NN, Artificial Neural Network—ANN, Decision Tree—DT, Support Vector Machine—SVM, random forest, among others. Within the second one, we can find Convolutional Neural Networks (CNN) [6,7,8], Constructive Deep Neural Network (CDNN) [9], Deep Neural Network (DNN) [10], a Deep Belief Network (DBN) [11], a Deep Boltzmann Machine (DBM) [12] as well as others. For the third category, according to Reference [4], we can find Fuzzy Expert Systems (FES), the Fuzzy Set Theory (FST), the Fuzzy Inference Systems (FIS), the Adaptive Neuro-Fuzzy Inference Systems (ANFIS), Fuzzy Neural Networks (FNN), fuzzy cognitive maps, and more.

Decision Support Systems (DSSs) are, therefore, solutions that serve the management in their decision-making process. DSS, by default, comprise interactive features to aid enough data and model analysis with the intent to classify and resolve predicaments as well as present resolutions [13]. According to Turban et al. [14], all DSS comprises four standard components: information, model, knowledge, and user interface management sections. The first component Information or Data administration focuses on the storage, manipulation, and maintenance of necessary information. The sources include either one or a combination of the following: The primary source of information, external sources, and personal information. The second component, which is the model, refers to the analytic tool that is the part responsible for the analysis. It includes financial, statistical, management science, or other quantitative models that support data analysis as well as system maintenance. The third component is the knowledge-based (KB) management subsystem. It provides information about data and their relationships, particularly complex data. It comprises possibilities, alternatives, rules, and methods for evaluating options to arrive at a decision. The fourth element is the user interface subsystem. The sole intent of the user interface is to present a mechanism within which the user and the DSS may communicate. DSS is interactive and, therefore, requires massive user communication [14].

For Clinical Decision Support Systems, many predictive or classification algorithms have been implemented to diagnose different diseases [15]. Among them, we can find works using fuzzy rule miner [16], Constructive Deep Neural Network [9], Support Vector Machines [17,18], ANFIS [19,20], genetic algorithms [21], random forest [22,23], Decision Trees [24,25], k-NN [24], and more. Most of these intelligent systems have their algorithms for “learning” from the data. For example, artificial neural networks “learn” using layers (inputs, hidden and output layers) and the Back Propagation—BP algorithm. Their learning process is made through the adjustment of weights and bias on every single layer. Hybrid techniques use the learning strength of the mentioned technique and adjust the parameters of other intelligent systems. For example, a neuro-fuzzy system uses the potential of artificial neural networks to adjust the parameters of a Sugeno-type fuzzy inference system.

For the development of intelligent systems using fuzzy logic, two primary components are needed including the knowledge database and the knowledge rule base. Mamdani-type fuzzy logic does not have an algorithm to “learn” their knowledge components (Database and rule base) from the data. It means that this type of fuzzy logic must be carried out manually with a high subjective element, that is, it is the modeler or specialist who determines the number of the fuzzy sets for each input and output variable establishing the ranges between these sets. The modeler or specialist also defines the set of rules that the fuzzy inference system will have. For this reason, the whole proposition of the system becomes subjective.

Another critical component that we need to point out is the rules number in the decision support system. This component is influenced by the number of variables that are required to provide the best performance. This component is known as a feature selection. For this, the modelers can use pre-processing techniques to help establish these characteristics. However, it would be time to add one more component to the proposed framework, which can increase the system’s processing time.

One problem about the learning process among some computational intelligence techniques is that they are considered “black-boxes” and there is no transparency in the training process. A decision support system based on an artificial intelligence technique must have the ability to explain the decision made and the process used for it. For the reasons above, it is essential to look for a fast and effective alternative that could “learn” from the data, and automatically generate the Mamdani-type fuzzy inference system transparently, mapping the interactions between variables (inputs) and the relationships with the output variables. Thus, the primary aim of this work is designing, implementing, and validating a framework for the development of data-driven Mamdani-type fuzzy Decision support systems using clustering and pivot tables to gather the mentioned components that are being obtained manually and subjectively.

As mentioned before, clinical decision support systems help clinicians and practitioners for decision-making about diagnosing some diseases. For this work, we selected three medical fields for the implementation and validation of the proposed framework: Breast cancer, Wart tumors, and Caesarean section.

Breast cancer is the most common and deadly cancer for women [26]. The symptoms may vary from person to person. However, this cancer has the following symptoms: lump in the breast, changes in breast shape, skin dimpling, orange like a scaly patch of skin, and more [27]. Approximately 40,920 deaths from invasive female breast cancer are predicted in the United States by the American Cancer Society (ACS) [28]. According to Reference [29], the Wisconsin Breast Cancer dataset was collected from the patients of University of Wisconsin-Madison Hospitals. The number of observations of the dataset is 699 data pair. The dataset has missing values. It is comprised of 458 benign cases and 241 malign cases. The descriptive statistics of the dataset can be found in Onan [26]. The input variables are 9, and the output variable is 1 (Breast cancer classification—benign and malignant).

The attributes of this dataset are [29]:

Attribute numberDomain1. Clump Thickness—CT1–102. Uniformity of Cell Size—UCSi1–103. Uniformity of Cell Shape—UCSh1–104. Marginal Adhesion—MA1–105. Single Epithelial Cell Size—SECS1–106. Bare Nuclei—BN1–107. Bland Chromatin—BC1–108. Normal Nucleoli—NN1–109. Mitoses—Mi1–1010. Class:(2 for benign 4 for malignant)

The literature shows that there are a lot of research studies about the breast cancer diagnosis using this Dataset (WBCD). Among the most recent works, we can find the followings: Liu, Kang, Zhang, and Hou [6] who reported that they were seeking to identify ways to improve the classification performance for this dataset based on convolutional neural networks (CNN). Their results were 98.71% for classification accuracy, a sensitivity of 97.60%, and a specificity of 99.43% for WBCD. The partition data method used by the authors was the five-fold cross-validation. Karthik et al. [10] used deep neural networks for the classification task and reported a classification accuracy of 98.62%. The dataset was partitioned using random sampling. Abdel-Zaher and Eldeib [11] used a deep belief network unsupervised path followed by a back propagation supervised path. Their results were 99.68% for classification accuracy, 100% for Sensitivity, and 99.47% for specificity. The used data partition method was random sampling. Onan [26] presented a classification model based on the fuzzy-rough nearest neighbor algorithm, consistency-based feature selection, and fuzzy-rough instance selection. This model obtained a classification accuracy of 99.71%, a sensitivity of 100%, and a specificity of 99.47%.

Regarding the Coimbra breast cancer, the dataset was collected from women newly diagnosed with breast cancer from the Gynecology department of the University Hospital Center of Coimbra between 2009 and 2013 [17]. This dataset comprises 116 instances (64 patients and 52 healthy volunteers) and nine input variables. The goal is to predict the presence of breast cancer in women. The descriptive statistics of the clinical features are presented in Table 1.

For the Coimbra Breast cancer dataset, Patrício, Pereira, Crisóstomo, Matafome, Gomes, Seiça, and Caramelo [17] used the Support Vector Machine, logistic regression, and random forest as classifiers. The authors used four input variables (glucose, resistin, age, and BMI) as predictors for the presence of breast cancer in women. The best results were obtained for SVM. Sensitivity results ranging between 82% and 88% and specificity ranging between 85% and 90%. The 95% confidence interval for the AUC was [0.87,0.91]. This study used 20-fold Monte Carlo Cross-Validation through 100 iterations. Polat and Sentürk [30] proposed a new machine learning-based hybrid method. They combined the normalization, feature weighting, and classification for the same dataset. The hybrid approach achieved 91.37% for accuracy.

Warts are benign tumors caused by infection with human papillomavirus (HPV) and can be categorized into three types: Cutaneous, Epidermodysplasia Verruciformis (EV), and Mucosal [25]. According to Al Aboud and Nigam [31], this medical problem affects approximately 10% of the population. The authors state that, in school-aged children, the prevalence is as high as 10% to 20%. The literature shows that there are two treatments: cryotherapy and immunotherapy. Many studies have been done to predict the appropriate treatment for each symptom. Among these works, we can find the followings: Khozeimeh, Alizadehsani, Roshanzamir, Khosravi, Layegh, and Nahavandi [19] identified appropriate treatment for two common types of warts (plantar and common), and they tried to predict the responses of two of the best methods for the treatment using a Fuzzy logic rule-based system. This study was conducted on 180 patients, with plantar and common warts, who had referred to the dermatology clinic of Ghaem Hospital, Mashhad, Iran. In this study, 90 patients were treated by the cryotherapy method with liquid nitrogen and 90 patients with the immunotherapy method. The selection of the treatment method was made randomly. The original first method dataset consists of seven features (six inputs and one output). The second one has eight features (seven inputs and one output variable). The classification attribute (label) is a response to treatment. The observed features in the clinical experiments are presented in Table 2.

The results show that the classification accuracy for immunotherapy and cryotherapy was 83.33% and 80.7%, respectively. As a data partition method, the authors used 10-fold cross-validation. Nugroho, Adji, and Setiawan [23] combined the two datasets to produce a single prediction method. As classifiers’ methods, they used the C4.5 algorithm combined with Random Forest Feature Weighting (RFFW) (C4.5+RFFW) to select the relevant features to improve accuracy. According to the results, the single prediction increases the classification accuracy to 87.22%. The authors used 10-fold cross-validation. Basarslan and Kayaalp [24] applied Naïve Bayes, C4.5 decision tree, logistic regression, and the k-nearest neighbor as classifiers for the dataset. The authors used 10-fold cross-validation. The results show that the C4.5 decision tree obtained the best performance for both datasets. The classification accuracy for cryotherapy dataset was 93.3% (raw data) and 95.4% (after correlation). For the immunotherapy dataset, the classification accuracy was 82.5% for raw data, and 84% after correlation. Jain, Sawhney, and Mathur [22] applied random forest (RF), binary gravitational search algorithm (BGSA), and the hybrid between these two techniques. Their results show that the highest classification accuracy for both datasets was 88.14%, and 94.81% for immunotherapy and cryotherapy, respectively, using BGSA + RF.

According to the World Health Organization (WHO) [32], the cesarean sections are associated with short-term and long-term risk, which can extend many years beyond the current delivery, and affect the woman’s health, her child, and future pregnancies. These risks are higher in women with limited access to comprehensive obstetric care. According to Reference [33], the mean caesarian delivery rate in the United States of America for 2017, was 32% for all births, showing a high percentage of use for this medical procedure, due to the international healthcare community that has considered the ideal rate for cesarean sections to be between 10% and 15% [32]. The Caesarean section dataset is comprised of five input variables and one output variable, and 80 instances. The dataset was collected using pregnant women’s information and the referred delivery in the Tabriz health center. Gharehchopogh et al. [34] worked with a C4.5 Decision Tree algorithm. The authors did not mention the data partition method. Their results for classification accuracy was 86.25%. Amin and Ali [35] utilized k-nearest neighbor, Support Vector Machine, Naïve Bayes, logistic regression, and random forest as classifiers. The authors did not mention the used data partition method. Their result is classification accuracy of 95% obtained for random forests and k-NN.

In all cases, it is critical to developing some tools (models) that assist in decision-making for early detection, appropriate therapy, and treatment [36]. For the reasons above, the main aim of this research is to develop classification models with high accuracy for the mentioned medical issues.

The originality of the current framework could be considered as follows:

- None of the feature extraction methods were applied before the cluster’s analysis.
- None of the machine learning algorithms was used or applied.
- Clustering was the unique data mining technique that we used.

The principal contributions of this work could be considered as follows:

- We present a novel method for knowledge discovery through an easy extraction of the knowledge database and knowledge rule base for the development of Mamdani-type Fuzzy Inference systems.
- Using clustering and pivot tables in classification problems allows knowledge discovery through patterns’ recognition. These two tools can increase the likelihood of reducing input variables (feature extraction), which simplifies the decision-making process.
- The use of the two tools could be considered a deep machine learning algorithm.
- All the algorithms were tested in real-world problem datasets (Appendix A, Appendix B, Appendix C, Appendix D, Appendix E, Appendix F, Appendix G and Appendix H).

The remainder of this paper is organized as follows. Section 2 discusses the theoretical background. Section 3 shows the material and methods (including the proposed framework). Section 4 presents the research findings. Section 5 describes the discussion and Section 6 provides the final conclusions of this paper.

## 2. Theoretical Background

### 2.1. Fuzzy Sets Theory

Fuzzy set theory is recognized as the foundation of all fuzzy logic methods [4]. Zadeh [37] proposed the fuzzy set theory as an extension of the classical set theory to model sets whose elements have degrees of membership [38].

The fuzzy set theory offers the tools to successfully characterize human expert knowledge [39]. According to Reference [38], a linguistic value denotes a label for knowledge representations that have meaning determined by its degree of the membership function. Fuzzy Rule-Base Systems (FRBs) are the most fruitful developments of this field [38].

FRBS can be explained as a type of rule-based system in which fuzzy sets in combination with the fuzzy logic are used to describe an expert knowledge based on the goal of the study and to model the relations between input and output variables to overcome the current inherent uncertainty knowledge [40]. The rules of this kind of system are frequently characterized as “IF…THEN” statement and each rule can be defined as a fuzzy conception. Fuzzy rules permit to successfully categorize data having non-axis-parallel decision limits, which is difficult for conventional attribute-based methods [41].

Fuzzy rule-based systems were developed in two types of approach such as Mamdani [42] and Takagi-Sugeno types [4,43]. The two approaches are going to be explained as follows.

#### 2.1.1. The Mamdani-Type Fuzzy Model

The Mamdani type model is a kind of fuzzy relational model, where the relationship IF-THEN represents each rule. It is also called a linguistic model because both the antecedent and consequent are fuzzy propositions [44]. Its structure is developed manually. The output of the Mamdani type model is a fuzzy membership function based on the rules created during the modeling process.

Mathematically and linguistically, it can behave as follows.

If *x* is *A* and *y* is *B*, then *z* is *C* (1)

where *x* and *y* are the system input variables, *z* is the system output variable, *A* and *B* are antecedent membership functions, and *C* is a consequent membership function.

Generally, software programs for the implementation of this type of model use the Centroid method for defuzzification, which can be considered a weighted average where the weights are represented by μA (xi), which indicates the degree of membership of the value xi with the concept modeled by the fuzzy output set A, and which, in its compound shape, is calculated by the equation below.

(2) $$Z=\frac{\mu c(z)z\delta z}{\mu c(z)\delta z}$$

where Z is the consequent variable and µc(z) is the function of the composed shape. The result of the defuzzification process Z can be continuous or discrete [45].

**Figure 1:**
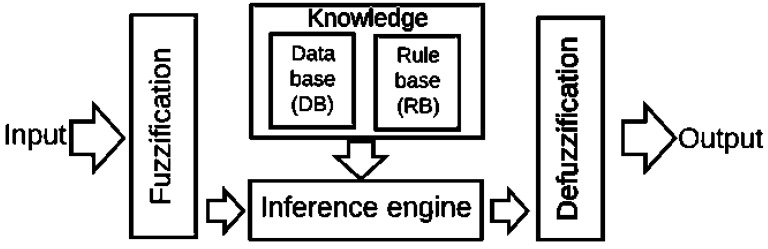
Mamdani-type fuzzy model components. Source: Reference [38].

The standard architecture for the Mamdani-type fuzzy model consists of four components (Figure 1):

Fuzzification: Converts the crisp inputs into linguistic values.

Knowledge base: it comprises a database and a rule base. The first one (DB) includes the fuzzy set definitions, and the membership functions parameters. The second one (RB) comprises the fuzzy IF. THEN rules collections.

Inference engine: Achieves the reasoning operations on the appropriate fuzzy rules and input data.

Defuzzification: Produces crisp values from the linguistic values as the results.

#### 2.1.2. The Sugeno-Type Fuzzy Model

The Sugeno type model [43]: for a system with two input variables and one output variable, the system is as follows.

If *x* is *A* and *y* is *B* then *z* = *f* (*x, y*) (3)

where *x* and *y* are the input variables, *z* is the output variable, *A* and *B* are antecedent membership functions, and *f* (*x*, *y*) is a crisp function in the consequent. Usually, this function is a polynomial of the input variables *x* and *y*. As an example, it can be cited as the case of the first-order polynomial, which is expressed as follows.

*Z = p*_1_*x + q*_1_*y + r*_1_ (4)

Defuzzification is expressed as a weighted average Z of the consequent functions.

(5) $$Z\;=\;\frac{\Sigma wz}{\Sigma w}$$

where *w* is the rule firing strength and *z* is a consequent function output.

Output and results in the Mamdani approach appear as a fuzzy set, while, in the Sugeno approach, the crisp output appears in the form of the fuzzy value [46]. In the Sugeno-type, the obtained rule base is not so easy to interpret [38].

According to Reference [38], the rule base was initially derived from human experts through knowledge engineering processes. However, this approach may not be feasible when facing complex tasks or when human experts are not available. If the experts are not available, there are other ways to generate the FRBS model automatically from data. These ways use learning methods. Among them, we can find space partition-based methods [47], neural-fuzzy techniques [48,49], subtractive clustering methods, and the gradient descendent learning [50], genetic algorithms [51], etc.

### 2.2. Decision Support Systems (DSS)

Decision Support Systems (DSSs) are, therefore, solutions that serve management in their decision-making process. DSS, by default, comprise interactive features to aid enough data and model analysis with the intent to classify and resolve predicaments as well as present resolutions [13].

#### Decision Support Systems’ Components

According to Turban, Aronson, and Liang [14], all DSS comprises four standard components: information, model, knowledge, and user interface management sections.

The first component Information or Data administration focuses on the storage, manipulation, and maintenance of information necessary. The sources include either one or a combination of the following: The primary source of information, External sources, and Personal information.

The second component, the model, refers to the analytic tool that is the part responsible for analysis. It includes financial, statistical, management science, or other quantitative models that support data analysis as well as system maintenance.

The third component is the knowledge-based (KB) management subsystem. It provides information about data and their relationships, particularly complex data. It comprises possibilities, alternatives, rules, and methods for evaluating options to arrive at a decision.

The fourth element is the user interface subsystem. The sole intent of the user interface is to present a mechanism within which the user and the DSS may communicate. DSS is interactive and, therefore, requires massive user communication.

### 2.3. Fuzzy Sets Theory in Clinical Decision Support Systems

Fuzzy Clinical decision support systems (FCDSS) are developed to convert knowledge from experts based on fuzzy rules to improve decision-making [4]. For developing this kind of system, the Mamdani-type FIS are widely used [52,53]. Fuzzy decision support systems are used in different knowledge areas such as Medicine [53,54,55,56,57], Agriculture [58,59], Financial [60,61,62,63,64], Construction [65], Education [66], and more.

### 2.4. Clustering Approach

Clustering is a data mining technique that is used in the process of dividing a set of data or objects into a set of essential sub-classes [67]. Several clustering methods exist in the literature. Among them, we can find the following.

#### 2.4.1. K-Means Clusters

According to Manivannan and Devi [67], K-Means is a clustering algorithm used to classify or group the objects based on attributes/features that are partitioned into the K number of the group where K is a positive integer number. Each cluster is a collection of analogous elements, which may be exclusive to that group, but are similar to each other [68].

#### 2.4.2. Hierarchical—Ward Method

According to Malhat and El-Sisi [69], the Ward method is one of the hierarchical agglomerative clustering algorithms that have a wide range of applications in a variety of areas because of its ability to group similar compounds in the same cluster. Each cluster initially corresponds to an individual item (singleton) [70]. As clustering proceeds, each group may contain one or more details [69].

#### 2.4.3. Nearest Neighbor

The main rule in the nearest neighbor clustering is to identify categories of unknown data using the already established nearest neighbor data group [71]. The nearest neighbor technique is divided into two categories: (1) structureless and (2) structure-based [72]. Data are grouped into training data and sample data points in the first one. Distance calculation performed on the entire training data to the data sample if a point has the closest or minimum distance. Those points are expressed as the nearest neighbor [71].

#### 2.4.4. Pivot or Pivot Tables

According to Reference [73], a pivot table is an interactive way to summarize large volumes of data quickly. The pivot table can be used to analyze numerical data in greater detail and to answer unforeseen questions about these data. The pivot tables are specially designed to: Consult large amounts of data in many simple ways such as to expand and collapse the data levels to highlight the results and delve into the details of the summary data of the areas of interest. To filter, sort, and group the most useful and exciting subsets of data, as well as format them conditionally, so you can focus on the information you want, among other characteristics [73]. For more information about this topic, please refer to Reference [74].

## 3. Materials and Methods 

The proposed framework for the development of data-driven Mamdani-type fuzzy clinical decision support systems using clustering and pivot tables will be explained.

The framework is a compound of t13 activity steps. The first three levels (1:3—Table 3) contain a complete understanding of a specific domain as well as possible gaps of existing models for evaluation. The next steps belong to the previous levels to the iterative design and development of data-driven clinical decision support systems (Steps 4:5, Table 4). The followings steps contain the iterative design and construction of data-driven clinical decision support systems (levels 6:9—Table 5). Step 7 and eight consists of the knowledge of database creation. The next section Pivot tables (step 9) explain the use of this resource as a feature selection and the knowledge rule base creation, and the following steps (10:11—Table 6) refers to the implementation of the data-driven Mamdani-type fuzzy clinical decision support systems. The last level is the communication process.

## 4. Case Studies

For validating the mentioned framework, five case studies were developed. All datasets for these case studies were obtained from the UC Irvine Machine Learning Repository [87] to evaluate the framework implementation effectiveness.

### 4.1. Step 1. Identifying the Datasets

All selected datasets belong to classification problems. Among the datasets are the Wisconsin Breast Cancer Dataset (WBCD) [88,89], the Coimbra Breast Cancer dataset [17], the Wart treatment (Cryotherapy and Immunotherapy) dataset [19,20], and the Caesarean section dataset [34,35].

For the Wisconsin Breast Cancer dataset, it was collected from patients at the University of Wisconsin-Madison Hospitals. The instances number for this dataset is 699 data pair. It has missing values. It includes 458 benignant cases and 241 malignant cases. The descriptive statistics of this dataset are shown in Onan [26]. It comprises nine input variables, and only one binary output variable (benign or malignant class).

Regarding the Coimbra breast cancer, the dataset was collected from women newly diagnosed with breast cancer from the Gynecology department of the University Hospital Center of Coimbra between 2009 and 2013 [17]. This dataset comprises 116 instances (64 patients and 52 healthy volunteers) and nine input variables. The goal is predicting the occurrence of breast cancer in women. The descriptive statistics of the clinical features are shown in Patrício, Pereira, Crisóstomo, Matafome, Gomes, Seiça, and Caramelo [17].

The Wart treatment dataset identifies the proper treatment for two common types of warts (plantar and common) and predicting the responses of two of the best methods (cryotherapy and immunotherapy) for the procedure. According to Patrício, Pereira, Crisóstomo, Matafome, Gomes, Seiça, and Caramelo [17], this study was conducted on 180 patients, with plantar and common warts, who had referred to the dermatology clinic of the Ghaem Hospital in Mashhad, Iran. In the research, 90 patients were treated by the cryotherapy method with liquid nitrogen and 90 patients with the immunotherapy method. The selection of the treatment method was made randomly. The original first method dataset consists of seven features (six inputs and one output). The second one has eight features (seven inputs and one output variable). The classification attribute (label) is the response to treatment. The primary goal is to select the best treatment method, saving time for patients, reducing costs, and improving the quality of treatment. The observed features in the clinical experiments are shown in Patrício, Pereira, Crisóstomo, Matafome, Gomes, Seiça, and Caramelo [17].

The cesarean section dataset consists of 80 cases, and it is composed of five variables. The input variables are age, number of pregnant, delivery time, blood pressure, and the heart status. The output is a Boolean variable (yes or no cesarean). The complete dataset is shown in Reference [34].

### 4.2. Data Preparation (Crisp Inputs)

This step means that the data stored in the multiple data sources must be pre-processed [76]. For the WBCD case study, all input and output variables were selected. The data were processed because there are missing values. The character “?” was transformed to zero. Another change in the dataset was to change the number two to one for benign cases and the number four to two for malignant cases. For the Coimbra breast cancer and cesarean section datasets, all input variables were selected to estimate the best performance among the interactions between those input variables. For the wart treatment dataset, not all original input variables were selected because the authors applied feature selection for both datasets. For cryotherapy, the authors chose four input variables (age range, the time elapsed before treatment range, types of the wart, and surface area of wart range). For immunotherapy, the selected features were the time elapsed before treatment, induration diameter of the initial test, and types of warts [19,20]. For the cesarean section dataset, all the input variables were selected for the training. The input variables delivery time, blood pressure, and heart status had to be transformed into nominal variables.

Clustering was the pre-processing technique used for each dataset. The use of clusters will be explained in Section 4.7. [81].

### 4.3. Reviewing Existing Models

In this step, the main aim is to look for related works of the problems with the objective for discussing our results and to compare with the research found in the literature. For this reason, searches about each classification problem were developed. The used indexed databases for this purpose were Science Direct, Scopus, Google Scholar, and Web of science. The results of these searches will be shown in the results and discussion sections.

### 4.4. Evaluating the Number of the Optimal Clusters

For this stage, pivot tables were applied for each classification problem. Table 7 shows an example of the optimal number of groups for each variable (input and output) for breast cancer datasets. The first row represents the number of rows that a pivot table throws when it is applied. This number represents the number of fuzzy sets that the inference systems could have if the modeler wants to make an inference system with all the values for each variable. However, some numbers are higher than 20 fuzzy sets. It refers that the rules number is going to be huge. For avoiding this problem, the recommendation is to apply the rounded square root. Doing this, the fuzzy sets number and the rules number for the data-driven fuzzy inference system decrease.

### 4.5. Setting the Number of the Clusters (Minimum and Maximum)

For all the case studies, the minimum number of clusters was 2. However, the minimum amount can be selected by the user. The maximum number of clusters for each classification problem were shown in the previous section. This means that the optimal number of groups was selected as a maximum.

### 4.6. Random Permutations

For all datasets, the inputs and outputs were randomized and permuted when applied the proposed algorithms (Appendix A, Appendix B, Appendix C, Appendix D, Appendix E, Appendix F, Appendix G and Appendix H).

### 4.7. Cluster Analysis (Fuzzification Process)

For all the case studies, we calculated the optimal number of clusters for each input and output variable. After that, according to the clustering method choice, for each input and output variable, we applied the clustering method to the data. We used the hierarchical cluster (Ward method) for classifying each variable (Appendix A). The Euclidean distance was selected as the default. This algorithm is recommended for handling if the dataset is not bigger than 10,000 instances. If the dataset is higher than this value, the recommendation is to use the non-hierarchical clusters algorithm like K-means. The maximum clusters number for every input and output variable for each dataset were the same optimal clusters values. After carrying out the previous process, the main idea is to identify the minimum and maximum value (into the raw data) for each input and output variable cluster. These two values represent the b and c parameters into the Membership functions (MF) for each variable. We proceed to extract the ranges of the quantitative variables (minimum and maximum). At the same time, we obtained the type of membership function (trapezoidal or triangular). If the minimum and maximum values (raw data) belonging to the same cluster value are equal, then the MFs type will be triangular. Otherwise, it will be trapezoidal. With these two values (min and max), we can calculate the remaining parameters for each MF (a and c or d according to the MF type). With all these parameters, we obtain the knowledge base (see Appendix B).

### 4.8. Sampling: Cross-Validation

For all case studies, all datasets were trained using two kinds of the data partition. The first one was using random sampling, which was randomly divided into two subsets: training and validation. The percentages for all cases were 70% for training, and the remaining 30% for validation. The users can manually select these percentages (Appendix D). The second one was using cross-validation. For all selected datasets, the value of k-folds was 10 (see Appendix H). In this stage, we proceed to extract the rule base through the pivot tables (see Appendix C).

### 4.9. Pivot Tables

For all case studies, the unique tables command were used for the development of the following sub-stages.

#### 4.9.1. Combining Different Cluster Datasets

This step consists of making combinations between input variables and the sets of output variables using pivot tables. These combinations are made using the command “nchoosek” and “unique” for matrixes (see Appendix C). The first command returns a matrix containing all possible combinations of the elements of vector v taken k at a time [83]. In the case studies, the vector v consisted of the clusters of the input variables with their respective output variables, and the parameter k was the number of input variables that were chosen for feature extraction. The k value can be selected from two and up to the maximum number of input variables for each classification problem. For example, the Wisconsin breast cancer data set has nine input variables, if we use the command “nchoosek” n represents the vector with the number of each of nine variables , n = [1, 2, 3, 4, 5, 6, 7, 8, 9]. In addition, k represents the number of variables that we want to select. If we choose two variables as a k value, the number of combinations that we can obtain is a matrix of 36 × 2, which indicates each possible combination from two variables. If you choose three variables as a k value, the number of combinations will be a matrix of 84 × 3, and so on. The second command returns a copy of a dataset A that contains only the sorted unique observations [83]. In the case studies, the dataset A represents the clusters’ dataset used for training. In this stage, the weights and connections for the rule base are calculated or implemented.

#### 4.9.2. Setting the Fuzzy Rules for each Variable

This section is based on the previous one. The operations carried out with the use of the pivot tables one or several combinations can be used for making the rule bases for the Data-driven Mamdani-type fuzzy inference systems. Using the unique command, we guarantee that all rules are unique by avoiding rules duplication (Appendix C). Table 8 shows an example of some values obtained from the previous step. In Table 8, the columns correspond to the groups’ numbers formed by the inputs and output variable clusters. The rows correspond to the rules’ numbers found in the clusters’ datasets. The interpretation of the created rule base is as follows.

IFInp_Var 1 is MF1, AND Inp_Var 2 is MF2, AND Inp_Var3 is MF3, AND Inp_Var 4 is MF2, and Inp_Var 5 is MF1, THEN, Out_Var is MF2. IF Inp_Var 1 is MF2 AND Inp_Var 2 is MF2, AND Inp_Var3 is MF3 AND Inp_Var 4 is MF1, and Inp_Var 5 is MF1, THEN, Out_Var is MF2, and so on. Some cluster values do not have any rule. This means that, within the training dataset, there are no measured or observed values for this interaction between the input variables [29,82]. According to the authors, if these cases occur, the inference engine calculates these values by combining input values with their respective membership degree, by the minimum operator and then by superposition of the rules through the maximum operator [90]. For the case studies, the Center of Gravity method was selected as the De-fuzzification process, which considers all output possibilities and transforms the fuzzy set originated by inference into a numerical value as proposed by Tanaka [91] and Sivanandam et al. [92].

### 4.10. Elaborating the Data-Driven Mamdani-Type Fuzzy Decision Support System (Inference Engine)

For the case studies, the data-driven clinical decision support systems’ implementation were carried out in the MATLAB^®^ 2017 software [83]. Through the current methodology, we must have the following elements: definition of the linguistic variables (variables names), the knowledge rule base, the fuzzy sets number, and the membership function values (knowledge database) for each variable. In this step, the main objective is to put all the components mentioned above in order. The first step is to create a blank (new) fis file. Put a name to the created fis file. Define the defuzzification process (must choice among these options: “Centroid,” “som—small of maximum,” “mom—mean of maximum,” or “lom—large of maximum”). Next step is to put names and ranges for every input variable. Within each input, the variable to put name, type, and parameters for every membership function. Repeat the procedure with output variable(s). The next step is to put the knowledge rule base in order. For this step, we must have the antecedent part (input variables clusters), the consequent part (output variables clusters), weights (values between 0 and 1), and connections for every single rule (values between 1 and 2). The first value represents the AND connector, and the second one represents the OR connector. For all implemented datasets, the values for weights and connections were one, respectively. For more illustration about this process, please refer to Appendix F.

For the case studies’ implementation, the fuzzy logic designer or toolbox was not used. Instead of that, we construct the data-driven Mamdani-type fuzzy inference systems using the MATLAB command line through our algorithms (Appendix A, Appendix B, Appendix C, Appendix D, Appendix E, Appendix F, Appendix G, Appendix H and Appendix I). Because it is a data-driven fuzzy inference system, it must be iterative. For all the case studies using a random sampling method, the two subsets resulting from Section 4.8 were used. The maximum number of iterations was 3000. With the use of the “nchoosek” command mentioned in Section 4.9.1, the generated files number with the extension “*.fis” can vary from one up to thousands, depending from the of input variables number and the selected iterations number. For the second option of data partition (cross-validation), the k-folds value was 10, which indicates that the maximum number of iterations was the same value. In addition, the mentioned commands were used. The computers used for the algorithm’s implementation was an AMD A12-9720P Radeon R7, 12 compute cores 4C + 8G 2.70 GHz with 16.00 GB RAM. Appendix J shows a link for downloading the implemented files.

### 4.11. Evaluating the Fuzzy Inference System Performance (Defuzzification and Crisp Outputs)

For all the case studies, the system’s performance was measured through some of the following metrics: The Classification accuracy (ACC), sensitivity, specificity, Function Measure, Area under the curve, and Kappa statistics.

#### 4.11.1. Classification Accuracy

This is one of the most popular metrics in the classifier evaluation [26]. According to the author, this metric corresponds to the proportion of the number of true positives (*TP*) and true negatives (*TN*) obtained by the classification algorithms in the total number of occurrences, as given by Equation (6).

(6) $$\mathrm{ACC}=\;\frac{\mathrm{TP}+\mathrm{TN}}{\mathrm{TP}+\mathrm{FP}+\mathrm{FN}+\mathrm{TN}}$$

where *FP* represents false positives and *FN* represents false negative instances. The accuracy value shows the proximity of the measured result to the true value. This value describes the accuracy of the results of the classification process.

#### 4.11.2. Sensitivity

It is another common metric for the validation of classification algorithms. It characterizes the true positives rate, and it is calculated by using Equation (7).

(7) $$\mathrm{Sensitivity}=\;\frac{\mathrm{TP}}{\mathrm{TP}+\mathrm{FN}}$$

#### 4.11.3. Specificity

It is defined as the number of true negative classifications divided by all negative classifications. It is represented by Equation (8).

(8) $$\mathrm{Specificity}=\;\frac{\mathrm{TN}}{\mathrm{TN}+\mathrm{FP}}$$

#### 4.11.4. F-Measure

For obtaining this metric, two other metrics must be calculated: the precision and recall. Precision is the proportion of the true positives against all the positive results. Equation (9) represents this metric.

(9) $$\mathrm{Precision}=\;\frac{\mathrm{TP}}{\mathrm{TP}+\mathrm{FP}}$$

The recall is the proportion of the true positives against true positives and false negatives. It is represented by Equation (10).

(10) $$\mathrm{Recall}=\;\frac{\mathrm{TP}}{\mathrm{TP}+\mathrm{FN}}$$

According to Onan [26], the F-measure is the harmonic mean of precision and recall, as given by Equation (11). This metric measure takes on values from 0 to 1. If the value of the F-measure is closer to 1, the better the classification algorithm is.

(11) $$F-\mathrm{Measure}=\;\frac{2\times \mathrm{Precision}\times \mathrm{Recall}}{\mathrm{Precision}+\mathrm{Recall}}$$

#### 4.11.5. Area under the Curve

It is equal to the likelihood that a classifier algorithm will rank a randomly chosen positive instance higher than a randomly chosen negative one. It represents the value of sensitivity and specificity with a boundary value of 0 to 1 [23]. According to Reference [93], this metric value can be categorized as follows.

- Between 0.90–1.00 = excellent classification;
- between 0.80–0.90 = good classification;
- between 0.70–0.80 = fair classification;
- between 0.60–0.70 = poor classification;
- between 0.50–0.60 = failure.

## 5. Experimental Results

For the validation of the performance of the data-driven Mamdani-type fuzzy Clinical decision support systems using clusters and pivot table, we compared our results with other models obtained from the literature (Section 4.3). The selected models obtained good performance in the same classification tasks. All the cited authors made comparisons between the proposed models by them and other classification algorithms (data mining or statistical techniques). All results show the performance using the two kinds of data partition (random sampling and cross-validation). The results showed in this section for the cases using cross-validation indicate the average results of the individual 10-fold.

Table 9 shows the models’ performance results for the Wisconsin breast cancer dataset. Table 10 presents the results for the Coimbra breast cancer datasets (CBCD). Table 11 and Table 12 present the results for wart treatment datasets, and Table 13 shows the models’ performance results for the cesarean section dataset.

## 6. Discussion

In this research study, a framework for the development of data-driven Mamdani-type fuzzy clinical decision support systems was proposed. With the objective for validating the results, different case studies were chosen and compared with other studies found in the literature. The case studies were carefully chosen because they are recent, and the authors compared their results with other previous research papers with other computational intelligence, statistical or data mining techniques, obtaining good performance in the classification tasks using the same selected datasets.

Regarding the Wisconsin Breast Cancer Dataset (WBCD). For the WBCD, the best performance from the mentioned authors in Table 9, belong to Onan [26], and Abdel-Zaher and Eldeib [11]. The first author used a classification model based on the fuzzy-rough nearest neighbor algorithm, consistency-based feature selection, and fuzzy-rough instance selection for a medical diagnosis. The second author proposed integration between Wavelet Transformation (WT) and Interval Type-2 Fuzzy Logic Systems (IT2FLS) with the aim to cope with both high-dimensional data challenge and uncertainty. Despite these mentioned results, it can be observed that none of the research works mentioned above reach the accuracy obtained in the current study with the specified number of variables (five). The proposed models with five variables exhibited the best performance indices related to the Wisconsin Breast Cancer dataset, which surpasses the results of advanced techniques (deep learning) such as Deep Belief Network, Deep Neural Network, and Convolutional Neural Networks. The achieved results for the metrics: sensitivity, specificity, F-measure, and Kappa statistics are closer to 1, which indicates a robust fit between the predicted classified data and the observed data. The area under the curve for this dataset is between 0.90 and 1.0, which means an excellent classification task [93]. The selected variables for both data partition methods were: Uniformity of Cell Size (UCSi), Marginal Adhesion (MA), Single Epithelial Cell Size (SECS), Bare Nuclei (BN), Normal Nucleoli (NN), which indicates that it is not necessary to make the mitosis process and reduces the processing time for the diagnosis and accelerates a possible treatment.

Similar to previous cases, for the Coimbra Breast Cancer dataset, the performance for this case study was higher than the obtained works from the literature. All metric values for both data partition methods are closer to 1, and the Kappa statistic is higher than 0.80, which represents a strong agreement between the classification predictions and the observed dataset. The selected variables for random sampling were all variables such as for cross validation, which were Body Mass Index—BMI (kg/m^2^), Glucose (mg/dL), Insulin (µU/mL), Homeostasis Model Assessment—HOMA, Leptin (ng/mL), Adiponectin (µg/mL), Resistin (ng/mL), and Monocyte Chemoattractant Protein 1 MCP.1. The authors concluded that, using four variables (Age, BMI, glucose, and Resistin), they could predict the presence of breast cancer in women with sensitivity ranging between 82% and 88% and specificity ranging between 85% and 90%. Our results are higher than those proposed by the mentioned authors.

Regarding the Wart treatment (cryotherapy dataset) presented in Table 11, it can be observed that the evaluation metric values obtained by the proposed framework (DDFCDSS) performs excellently with the highest classification accuracy (100%) for both data partition methods. This value is the most top predictive classification performance among the reported classifiers obtained from the literature. The values for the other metrics are 1, which represents an excellent fit between the classified data and the observed data. The selected variables for these results are: age, time elapsed before treatment (months), number of warts, type of warts (count), and surface area (mm^2^).

Like the previous case, for the wart treatment immunotherapy dataset (Table 12), for both data partition methods, the evaluation metrics values are the highest for the predictive performance among the other classifiers mentioned in the literature. In this case, the random sampling method worked with only the three variables (time elapsed before treatment, duration diameter of the initial test, and type of warts) mentioned by Reference [19]. For the cross-validation method, the future extraction proposed in this framework was used. Five variables were selected. The best performance for this training process was obtained for the following variables: age, time elapsed before treatment (months), type of warts (count), surface area (mm^2^), and induration diameter of the initial test (mm).

For the cesarean section dataset, the classification accuracy of our framework for the random sampling method is equal to the results shown by Reference [35]. The authors used different supervised machine learning classifiers such as support vector machines, naïve Bayes, logistic regression, k-nearest neighbor, and random forest. The classification accuracy for the best two models (the last two techniques) was 95%. For the cross-validation method, our results were higher than the results shown by Reference [34]. However, these results were lower than the results shown by Reference [35]. Our approach got an area under the curve of 0.9565 for random sampling and 0.93 for cross-validation methods, which indicates an excellent classification task. The Kappa statistics value is also very close to 0.90 for both data partition methods, and they were higher than 0.80, which represents a good fit between the observed data and the predicted ones. These results show that our algorithms can compete with other supervised machine learning classifiers.

The results shown in all mentioned tables (Table 9, Table 10, Table 11, Table 12 and Table 13) indicate that the proposed framework for the development of data-driven Mamdani-type fuzzy clinical decision support systems can be used as a reliable approach for helping in the decision-making processes.

A possible explanation for all these results is that pivot tables can characterize every single rule among the proposed layers for each selected problem. In fuzzy inference systems, this is a fascinating result because one rule can significantly affect (positively or negatively) the other rules’ performance. Furthermore, pivot tables can be able to find the non-linear function into the training data set without using any mathematical concept like descendent gradient, least square, or other optimization function. This result is significant because most of the used classifiers utilize some of these optimization functions.

According to these results, it could be demonstrated that the knowledge database and the rule base can be extracted transparently. In this way, the final decision support system shows the interaction or relationships between inputs and output variables. The novelty of this work can be considered as follows. We do not need to calculate any weights, bias adjustment, or optimization function to determine these relationships. The pivot tables are effectively able to capture relevant high-level abstraction and characterize a training data set, which offers insights about how to arrive at a conclusion or decision.

## 7. Conclusions

This paper presented a detailed framework for the development of data-driven Mamdani-type fuzzy clinical decision support systems using clusters and pivots tables. The main contribution of this study is two-fold. First, the methodology was successfully employed to extract significant features from low-dimensional and high-dimensional datasets. Second, clusters and pivot tables can help obtain the knowledge base, and the pivot table can help extract the fuzzy rule base from the inputs/outputs data in a natural way. These two components represent the knowledge-based management subsystem in a Decision Support System, which provides information about data and their relationships helps to arrive at decisions. In addition to the above, we can conclude that:

1. The developed data-driven Mamdani-type fuzzy inference models (experimental results) indicate that it can be used as a classifier because they obtained very promising evaluation metrics. The Performance metrics, specificities, sensitivities, F-Measures, areas under the curve, and Kappa statistics for all datasets: Wisconsin and Coimbra Breast Cancer Datasets, wart treatment immunotherapy and cryotherapy) and the Cesarean section dataset were higher, which is very close to 1 and allows an excellent performance.

2. The experimental results indicate that all the developed data-driven Mamdani-type fuzzy clinical decision support systems can be used as tools for automated diagnosis or decision-making processes.

3. The current framework provides a real pattern for the development of data-driven Mamdani-type fuzzy clinical decision support systems for classification problems.

4. We could state that this framework could be considered as a deep learning technique because it is composed of various processing layers to learn representations of data with multiple levels of abstraction [96] and use a cascade of multiple layers of non-linear processing units for feature extraction and transformation. Each successive layer uses the output from the previous layer as input.

Several cons may exist in this research. First, this framework only worked with two kinds of membership functions: trapezoidal and triangular. An idea as future work is working with other membership functions (i.e., Gaussian, gbell, smf, etc.). Second, this framework only worked with two kinds of clusters: The Ward method and k-means. The idea for other future works is to use different types of clusters approaches or methods like the *k*-Nearest Neighbors (k-NN) or enhanced k-NN proposed by Reference [97], Fuzzy C-means, and others. Third, the number of rules can vary depending on the problem. It depends on the cluster number sizes and the number of repetitions of the variable’s values (number of rows for the pivot tables). An idea for future work is to use pivot tables for optimizing the number of rules for every data-driven Mamdani-type fuzzy Decision Support system developed with this framework.

The main future work is to apply this framework in two fields: big datasets (big data) and regression problems. In addition, we would like to use it for the development of data-driven Mamdani fuzzy clinical decision support systems for imaging problems.

## Figures and Tables

**Table 1:** Features in the original Coimbra Breast Cancer dataset.

Feature	Attribute	Patients	Controls	*p*-Value
1	Age (years)	53.0 (23.0)	65 (33.2)	0.479
2	BMI (kg/m^2^)	27 (4.6)	28.3 (5.4)	0.202
3	Glucose (mg/dL)	105.6 (26.6)	88.2 (10.2)	<0.001
4	Insulin (µU/mL)	12.5 (12.3)	6.9 (4.9)	0.027
5	HOMA	3.6 (4.6)	1.6 (1.2)	0.003
6	Leptin (ng/mL)	26.6 (19.2)	26.6 (19.3)	0.949
7	Adiponectin (µg/mL)	10.1 (6.2)	10.3 (7.6)	0.767
8	Resistin (ng/mL)	17.3 (12.6)	11.6 (11.4)	0.002
9	MCP-1 (pg/dL)	563 (384)	499.7 (292.2)	0.504

**Table 2:** Features in the original immunotherapy and cryotherapy datasets.

Immunotherapy			
Feature	Attribute	Type	Values
1	Sex	Numeric	41 male and 49 females
2	Age	Numeric	15–56
3	Time elapsed before treatment (months)	Numeric	0–12
4	Number of warts	Numeric	1–19
5	Type of warts (count)	Numeric	47 common, 22 plantar, and 21 both
6	Surface area (mm^2^)	Numeric	6–900
7	Induration diameter of initial test (mm)	Numeric	5–70
8	Response to treatment	Nominal	Yes or no
**Cryotherapy**			
1	Sex	Numeric	47 male and 43 females
2	Age	Numeric	15–67
3	Time elapsed before treatment (months)	Numeric	0–12
4	Number of warts	Numeric	1–12
5	Type of warts (count)	Numeric	54 common, 09 plantar, and 27 both
6	Surface area (mm^2^)	Numeric	4–750
7	Response to treatment	Nominal	Yes or no

**Table 3:** First design steps for the framework for the development of data-driven Mamdani-type CDSS.

DDMTFCDSS Activity Steps	Activity Description
**Identifying the problems and evaluating the complexity of the specific domain**	
1. To identify the dataset.	This stage is related to identifying the source of the collected data to make the fuzzy inference system. These data usually belong to experiments that seek to observe the behavior of some dependent variables through the interaction of independent variables. Generally, the first variables are known as output variables and the second are known as input variables. This section describes the context and the adopted methodology to obtain the database that will serve as an input to work with the other framework components.
2. Data Preparation (Crisp inputs).	This step, according to the methodology proposed by Palit and Popovic [75] and modified by Cavalcante et al. [76], means that the data stored in multiple data sources (Spreadsheets, Data Bases—DBs, Comma-Separated Values—CSV, Enterprise Resource Planning—ERP, Customer Relationship Managers—CRM, Material Requirements Planning—MRP, among others) must be pre-processed [76]. The first part of this phase is to define the input and output variables that will be used for modeling. The pre-processing is a procedure where datasets (input(s) and output(s)) are prepared to be processed by the data mining technique (clusters) and the computational intelligence (fuzzy system). For doing that, in the literature, some pre-processing mechanisms used to improve the prediction or classification performance, among these, we can found Feature selection [77], Feature Extraction [78], de-noising, outlier detection [79], Time series segmentation [80], and Clustering [81]. Datasets must be normalized and structured. This can be done with the help of spreadsheet software like Microsoft Excel^®^ among others.
3. Reviewing existing models.	In this stage, an academic and scientific search of the different works related to the problem is carried out. For this, different indexed databases such as Scopus, Science Direct, Web of Science, Scielo, Google Scholar, ACM, etc. are used.

**Table 4:** Previous steps to the iterative design for the framework for the development of Data-driven Mamdani-type CDSS.

DDMTFCDSS Activity Steps	Activity Description
4. Evaluating the optimal number of clusters.	This stage pretends to find the optimal number of clusters for each one of the input and output variables. It determines a reference pivot number for adding or removing groups depending on whether you want to reduce or increase the number of fuzzy sets for each variable. This cluster number will indicate the same amount of fuzzy sets for the selected variable. If the variables are input, then you can determine the number of rules that the fuzzy system will have through the interaction between them. If the variables are output, then the number of the cluster will be the number of the fuzzy set that will have that variable, but it will not affect the rule set number. For the reasons above, it is important to have this optimal value of clusters, because it will give us an initial idea of how many rules and how many fuzzy sets we must have at maximum for each variable.To determine the optimal number of clusters, for each variable (input and output(s)), we applied pivot tables to establish the maximum number of clusters that could have each one. The pivot table makes a unique table (without repetitions of values). If the optimal number of clusters mentioned above is greater than 20, it is recommended to calculate the square root of this value and take that value as the optimal number of clusters. The recommended minimum number of clusters is two (2)—Appendix A.
5. Setting a number of clusters (minimum and maximum) according to the previous evaluation.	Determining the optimal number of clusters, we can have an idea of how many fuzzy sets and the number of rules we can have for the construction of the fuzzy system. Based on that optimal number of clusters, we can establish a range (minimum and maximum) in which the result of the previous section is within it. For example: If the result of the previous step threw five (5) clusters for the first input variable, we could set the range between minimum two (2) and maximum five (5). This is done to see the possibilities of reducing the number of clusters that can directly affect the number of rules or, on the contrary, see if the performance with a higher number of rules can be improved. For this case, the principal idea is to optimize the effectiveness of the established fuzzy system with a reduced rules number—Appendix A.

**Table 5:** Iterative design steps for the framework for the development of Data-driven Mamdani-type CDSS.

DDMTCDSS Activity Steps	Activity Description
**Begin Iterative Design and Development of Data-Driven Clinical Decision Support Systems**	
6. Random permutations	This stage allows us to make random permutations into the selected dataset(s). This is made to avoid the same input and output(s) order and could be chosen different classes or attributes at the moment for choosing the two or three subsets for training, validation, and test through random sampling or cross-validation processes.
**Knowledge Database Creation**	
7. Cluster analysis: fuzzification process.	At this stage, the main idea is to classify the data values of the respective input and output variables, according to the criteria set out in the previous section. For the individual analyses, it is recommended to use at least one of the two types of algorithms recognized in the academic and scientific field. Non-hierarchical (K-means) and hierarchical clusters (nearest neighbor and the Ward method) can be used to evaluate the performance of each one of the algorithms through the next phase of the methodology—Appendix A. Depending on each variable, these results (clusters) could change for each case. The result of this stage is the knowledge representation or knowledge base for the fuzzy inference systems because the clusters represent the number of fuzzy sets (input and output range values or membership functions). See Appendix B.
8. Sampling: Cross-validation datasets.	This stage seeks to randomly divide the dataset into two or three sub-sets. The framework proposes two kinds of data partition. The first one consists of random sampling. The user can select two or three subsets (training, validation, and testing). Generally, the percentage for each one, could be: 70:30:0; 70:20:10; 70:15:15; 70:10:20, etc. The user chooses this percentage. See Appendix D. The user also chose the number of repetitions that want to have, for example, if the problem has three input variables. The recommendation is to make at least three repetitions, which increases the possibility of finding an optimal combination of the sets formed by the clusters by avoiding some set(s) outside of the learning process. The results of the two or three selected subsets are saved into a multidimensional array with the divided datasets. The second option for data partition is through the Cross Validation process. In this option, the dataset is randomly partitioned into *k* equal sized partitions (by default is 10 —the users can change this value). One partition is used for testing the performance of the system, whereas the rest of the partitions is used for the training process. This procedure is repeated *k* times to use each partition as a test subset exactly one time. In the end, a mean accuracy of the individual results is consolidated. All two processes are automatized. See Appendix H.
**Pivot Tables: Feature Selection and Knowledge Rulebase Creation.**	
9. Pivot tables	Knowing the number of the optimal clusters to each variable, we can calculate the rules number that the fuzzy system can have through the interaction between the input variables—Rulebase. According to Hernández-Julio, Hernández, Guzmán, Nieto-Bernal, Díaz, and Ferraz [29] and Hernández-Julio, et al. [82], this stage looks for a combination that allows capable results without having to reach the maximum number of input variables—Feature extraction. Doing this, it is guaranteed that the fuzzy system will be optimized in the number of variables (minimum) and the number of fuzzy sets for each variable, mainly in the output variables—knowledge discovery. In this phase, the following sub-phases are done (Table 4)—Appendix A and Appendix C.

**Table 6:** Pivot tables substages and decision support systems’ implementation steps.

DDMTFCDSS Activity Steps	Activity Description
9.1 Combining different input variable cluster datasets.	The objective in this stage is to make combinations between input variables clusters datasets generated in the previous step, to find the best performance. For doing this, we can use the command nchoosek from any matrices laboratory software—Appendix C.
9.2 Establishing the fuzzy rules.	This section is based on the previous one. If the operations carried out with the use of the pivot tables find one or several combinations that guarantee good results (not clusters overlapping or minimum differences between the values), it proceeds to make the rules base of the fuzzy system. This is done by the recommendations of the previous section (using the command Unique). The rules will be easily detectable. To see an example refers to the case studies—Appendix C.
**Data Driven Mamdani-Type Fuzzy Clinical Decision Support System**	
10. Elaborating the Decision Support System based on a fuzzy set theory (Inference engine).	This stage refers to the implementation in specific software for the elaboration of fuzzy model systems such as Matlab^®^ [83], Xfuzzy^®^ [84], sciFLT^®^ [85], and more. For doing this, until the moment, we must have, through the current framework, the following elements: definition of the linguistic variables, the rules set, the sets number, and the membership function values of each variable. In this step, the modeler must know the environment of the software platform to work with each of the elements previously mentioned. The recommendation for this stage is that the modeler may need to manually adjust the values of the sets suggested by the methodology, until achieving the desired values (Appendix F).
11. Evaluating the fuzzy inference system performance (defuzzification and Crisp Outputs).	This stage aims to measure the designed and implemented system performance until this moment. For doing this, we must use the evaluation functions of each specific program and realize the simulations with the observed data and with the mean values of these or the test data subset (Appendix E). In this case, the recommendation is to try to perform the simulation and get the results in a table format, where you can realize some statistical calculations that allow us to evaluate the model’s performance. As part of the recommendation, for regression problems, within the calculations carried out by the system for each output variable, there are the following statistical indexes: absolute deviation, standard deviation, percentage error, a graph of coefficient of Correlation R, and a calculation of the coefficient of determination R2 (see Appendix E and Appendix G). Other statistical values can be calculated. However, they could be made in a spreadsheet with the results of the output variables observed and those predicted by the system. These calculations can be: standard error, Root Mean Square error (RMSE), regression coefficients (slopes), intercepts, and more. If the obtained model’s results suggest that the system has a good or excellent performance, the modeling process is finished, changes are saved, and the results are shown. For classification problems, the system’s performance evaluation could be measured through the following metrics: The Classification accuracy (ACC), sensitivity, specificity, Function Measure, Area under the curve, and Kappa statistics. These evaluation metrics are explained in detail in Reference [26] and Reference [86] with their respective formulae.
**End of Iterative Process**	
12. Communication.	This stage refers to the paper or documentation preparation. In this case, the modeler may show the results through a user manual or an academic and scientific journal article. If the main aim is to publish a journal article, the target population must be researchers or practitioners within the interest domain.

**Table 7:** Optimal clusters number for breast cancer datasets.

	Inputs									Output
	CT	UCSi	UCSh	MA	SECS	BN *	BC	NN	MI	
**Wisconsin Breast Cancer**										
Rows Number	10	10	10	10	10	11	10	10	10	2
Rounded Square root	10	10	10	10	10	11	10	10	10	-
**Coimbra Breast Cancer**										
	**Age**	**BMI**	**Glucose**	**Insulin**	**HOMA**	**Leptin**	**Adiponectin**	**Resistin**	**MCP-1**	
Rows Number	51	110	50	113	116	116	115	116	113	2
Rounded Square root	7	10	7	11	11	11	11	11	11	-

CT = Clump Thickness. UCSi = Uniformity of Cell Size. UCSh = Uniformity of Cell Shape. MA = Marginal Adhesion. SECS = Single Epithelial Cell Size. BN = Bare Nuclei. BC = Bland Chromatin. NN = Normal Nucleoli. MI = Mitoses. BMI = Body Mass Index. HOMA= HOMA-homeostasis model assessment for insulin resistance. MCP-1 = Monocyte Chemoattractant Protein-1. * indicates that there are missing values and were replaced by zero.

**Table 8:** Example of some values obtained from a unique table for the caesarean dataset.

Inp_Var 1	Inp_Var 2	Inp_Var 3	Inp_Var 4	Inp_Var 5	Out_Var
1	2	3	2	1	2
2	2	2	3	1	2
3	4	3	1	2	2
4	4	1	3	1	1
4	4	3	2	2	1
5	4	1	2	2	1
6	3	2	2	2	2

Inp_Var = Input variable. Out_Var = Output variable.

**Table 9:** Performance metrics obtained with our proposed framework and other classifiers obtained from the literature for WBCD.

	References									DDFCDSS—This Work	
Main Aspects		[29]	[11]	[10]	[9]	[94]	[6]	[26]	[95]	Five Variables (RS)	Five Variables (CV)
Num of Variables		3	9	-	6	9	9	7	3	5	5
Num of Rules or Hidden neurons/technique		39/FIS	DBN/4:2	DNN	DNN	SMO	FCLF and CNN	FRNN	WT and IT2FLS	192/DDFCDSS	233/DDFCDSS
Performance	Accuracy (%):	98.57%	99.68%	98.62%	96.2%:96.6%	72.70%	98.71%	99.72%	97.88%	**99.28%**	**99.40%**
	Sensitivity:	0.9793	1.000	-	-	-	0.976	1.000	0.9850	**0.9836**	**0.988**
	Specificity:	0.9891	0.9947	-	-	-	0.9943	0.9947	0.9650	**0.9978**	**0.998**
	F-Measure:	0.9793	-	-	-	0.71	-	0.9970	-	**0.9897**	**0.992**
	Area under curve:	0.9901	-	-	-	0.63	0.9816	1.000	0.9750	**0.9936**	**0.995**
	Kappa statistics:	0.9683	-	-	-	-	-	0.9943	-	**0.9842**	**0.987**

FIS: Fuzzy Inference System. DBN: Deep Belief Network. DNN: Deep Neural Network. SMO: Sequential Minimal Optimization. CNN: Convolutional Neural Network. FCLF: Fully Connected Layer First. FRNN: Fuzzy-Rough Nearest Neighbor. WT: Wavelet transformation. IT2FLS: interval type-2 fuzzy logic system. DDFCDSS: Data-driven Fuzzy Decision Support System. RS: Random Sampling. CV: Cross Validation. - which are not mentioned in the literature. Bold values indicate the best performance with fewer input variables.

**Table 10:** Performance metrics obtained with our proposed framework and other classifiers obtained from the literature for CBCD.

Main Aspects		Reference [17]						Reference [30]	DDFCDSS—This Work	
									CV	RS
Number of Variables		V1:V2	V1:V3	V1:4	V1-V5	V1:V6	V1:V9	V1:V8	V2,V3,V6,V8,V9	V1:V9
Number of Rules or Hidden neurons/technique		SVM						AdaBoostM1 and MAD	97	81
Performance	Accuracy (%):	-	-	-	-	-	-	91.37	**95.9%**	**94.0%**
	Sensitivity:	0.81:0.86	0.87:0.92	0.82:0.88	0.84:0.9	0.81:0.86	0.75:0.81	-	**0.937**	**0.901**
	Specificity:	0.7:0.76	0.78:0.83	0.84:0.9	0.81:0.87	0.8:0.86	0.78:0.84	-	**0.992**	**1.000**
	F-Measure:	-	-	-	-	-	-	0.914	**0.964**	**0.948**
	Area under curve:	0.76:0.81	0.82:0.86	0.87:0.91	0.86:0.9	0.83:0.88	0.81:0.85	0.938	**0.957**	**0.933**
	Kappa statistics:	-	-	-	-	-	-	82.76%	**0.917**	**87.6%**
	Precision:	-	-	-	-	-	-	0.919	**0.994**	**1.000**
	Recall:	-	-	-	-	-	-	0.914	**0.937**	**0.901**

CBCD: Coimbra Breast Cancer Dataset. V: Variable. SVM: Support Vector Machine. MAD: Mean Absolute Deviation. DDFCDSS: Data-driven fuzzy clinical decision support systems, - which are not mentioned in the literature. Bold values indicate the best performance.

**Table 11:** Performance metrics obtained with our proposed framework and other classifiers obtained from the literature for Wart treatment (Cryotherapy) dataset.

Main Aspects		References								This Work	
		[19]	[24]	[18]	[22]	[23]		[25]	[21]	RS	CV
Number of Variables		4	3	6 *	6	3/Merged dataset	3	2	6	4	4
Number of Rules or Hidden neurons/technique		ANFIS	NB, C4.5, DT, LR, K-NN	SVM	RF, BGSA + RF	C4.5 + RFFW		DT	J48, J48 + GA	46/DDFCDSS	52/DDFCDS
Performance	Accuracy (%):	80%	95.40%	85.46%	94.81%	87.22%	93.33%	94.4%	93.3%	**100.0%**	**100%**
	Sensitivity:	0,820	0.976	0.474	-	0.805	0.885	-	0.989	**1.000**	**1.000**
	Specificity:	0,770	-	0.958	-	0.908	0.980	-	0.130	**1.000**	**1.000**
	F-Measure:	-	0.933	-	-	0.799	0.919	-	0.989	**1.000**	**1.000**
	Area under curve:	-	-	-	-	0.830	0.617	-	0.988	**1.000**	**1.000**
	Kappa statistics:	-	-	-	-	-	-	-	0.977	**1.000**	**1.000**
	Precision:	-	0.937	-	-	-	-	-	0.989	**1.000**	**1.000**
	Recall:	-	-	-	-	-	-	-	0.989	**1.000**	**1.000**

ANFIS: Adaptive Neuro-Fuzzy Inference System, which is not mentioned in the literature. NB: Naive Bayes. DT: Decision Tree. LR: Logistic Regression. K-NN: K-Nearest Neighbor. SVM: Support Vector Machine. GA: Genetic Algorithm. C4.5: decision tree algorithm. * Both datasets. RF: Random Forest. BGSA: Binary Gravitational Search Algorithm. RFFW: Random Forest Feature Weighting. DDFCDSS: Data-Driven Fuzzy Decision Support System. RS: Random Sampling. CV: Cross Validation. - which are not mentioned in the literature. Bold values indicate the best performance.

**Table 12:** Performance metrics obtained with our proposed framework and other classifiers obtained from the literature for wart treatment (Immunotherapy) dataset.

Main Aspects		References								This Work	
		[19]	[24]	[18]	[22]	[23]		[25]	[21]	RS	CV
Number of Input Variables		3	3	6	6	3/Merged dataset	3	2	7	3	5
Number of Rules or Hidden Neurons/Technique		ANFIS	NB, C4.5, DT, LR, K-NN	SVM	RF, BGSA + RF	C4.5 + RFFW		DT	J48, J48 + GA	57/DDFCDSS	78/DDFCDSS
Performance	Accuracy (%):	83.33%	84%	85.46%	88.14%	87.22%	84.44%	90%	96.66%	**97.8%**	**97.8%**
	Sensitivity:	0.870	0.832	0.474	-	0.805	0.55	-	0.967	**0.986**	**0.973**
	Specificity:	0.710	-	0.958	-	0.908	0.9143	-	0.086	**0.947**	**1.000**
	F-Measure:	-	0.851		-	0.799	0.611	-	0.966	**0.986**	**0.986**
	Area under curve:	-	-		-	0.830	0.707	-	0.972	**0.966**	**0.947**
	Kappa statistics:	-	-		-	-	-	-	0.898	**0.933**	**0.93**
	Precision:	-	0.901		-	-	-	-	0.966	**0.986**	**1.000**
	Recall:	-	-		-	-	-	-	0.967	**0.986**	**0.973**

ANFIS = Adaptive Neuro-Fuzzy Inference System, which is not mentioned in the literature. NB: Naive Bayes. DT: Decision Tree. LR: Logistic Regression. K-NN: K-Nearest Neighbor. SVM: Support Vector Machine. C4.5: decision tree algorithm. RFFW: Random Forest Feature Weighting. GA: Genetic Algorithm. DDFCDSS: Data-driven Fuzzy Decision Support System. RS: Random Sampling. CV: Cross Validation. - which are not mentioned in the literature. Bold values indicate the best performance.

**Table 13:** Performance metrics obtained with our proposed framework and other classifiers obtained from the literature for a caesarean section dataset.

Main Aspects		References		This Work	
		[34]	[35]	RS	CV
Number of Input Variables		5	5	5	5
Number of Rules or Hidden neurons/technique		C4.5 DT/31 and 21 leaves nodes	k-nearest neighbors and Random forest	74/DDFCDSS	67/DDFCDSS
Performance	Accuracy (%):	86.25%	95%:95%	95%	93.4%
	Sensitivity:	0.8630	0.95:0.95	1.0000	0.934
	Specificity:	0.1090	0.037:0.052	0.8947	0.934
	F-Measure:	0.9460	-	0.9545	0.943
	Area under the curve:	-	0.995:0.994	0.9565	0.93
	Kappa statistics:	0.7281	0.8992:0.8977	0.8992	0.864
	Precision:	0.8860	0.955:0.950	0.9130	0.952
	Recall:	0.8630	-	1.0000	0.934

DT = Decision tree. DDMTFCDSS = Data-driven Mamdani-Type Fuzzy Clinical Decision Support System. RS: Random Sampling. CV: Cross Validation. - which are not mentioned in the literature.

## Appendix A. Pseudo-Code for the Implementation of the Fuzzification Function

**function [KnowledgeDataBase, totalmatrix, ranges, MFs] = Fuzzification (matrix, cluster_method, numinputs, numoutputs)**
numvariables = numinputs+numoutputs;
maxnumrows = 0;
for i = 1: numvariables
rowssize(i) = unique(matrix(:,i),’rows’,’stable’);%pivot tables
if rowssize > maxnumrows
maxnumrows = rowssize(i);
end
end
if maxnumrows > 20
maxnumrows = round(sqrt(maxnurows,0));
end
for i = 1: numvariables
if rowssize(i) > maxnumrows
optimaly = round(sqrt(rowssize(i),0));
rowssize(i) = optimaly;
else
optimaly = rowssize(i);
end

if cluster_method == ‘Ward’
clustering = clusterdata(matrix(:,i),’linkage’,’ward’,optimaly);%here we apply clustering methods
else
clustering = kmeans(matrix(:,i),optimaly);
end
totalmatrix = [matrix clustering];
[ranges,knowledgeDataBase,MFs] = knowledgeDB(maxnumrows,matrix(:,i),clustering,i);
end
end

## Appendix B. Pseudo-Code for the Implementation of KnowledgeDB Function

function [ranges,knowledgeDataBase,MFs] = knowledgeDB (maxnumrows,matrix,clustering,i)
calculate maximum from clustering;
for j = 1: maximum
k = find(clustering == j);
aA = matrix(k);
calculate min from aA; %this value is representing the value of b in the MF.
calculate max from aA; %this value is representing the value of c in the MF.
end
if min from aA == max from aA
MF(i) = ‘trimf’;
calculate the point_a. Point_c is the same value of max from aA or min from aA.
else
MF(i) = ‘trapmf’;
calculate the point_a and point_d for the Membership function.
end
knowledgeDataBase = [point_a Min(aA) Max(aA) point_d];
ranges = [min(aA) max(aA)];
MFs = MF;
end
end

## Appendix C. Pseudo-Code for the Implementation of KnowledgeRB Function

function [knowledgeRuleBase] = knowledgeRB(round,training, validation,testing,numvarinputs)
for a = 2:numvarinputs percent, it could be the same numvarinputs if you work with the same number of input variables.
numvars = a;
n = 1:1:numvarinputs;
combinations = nchoosek(n,numvars);
for i = 1:size(combinations,1)
for j = 1:size(combinations,2)
clustersinputsindices(i,j) = combinations(i,j) *2;
end
end
calculate the clustersoutputsindices;
for i = 1:size(combinations,1)
[RuleBaseInputs,ia,ic] = unique(training(:,clustersinputsindices(i,:)),’rows’,’sorted’); %pivot tables
RuleBaseOutputs = training(ia,clustersoutputsindices(i,:));
RuleBase = [RuleBaseInputs RuleBaseOutputs];
weightsandconnections = ones(size(RuleBase,1),2);
[knowledgeRuleBase] = [RuleBase weightsandconnections];
InferenceEngineandDefuzzification(combination,trainingdata,validationdata,testdata,knowledgeRuleBase,indicesinputs,indicesoutputs);
end
end
end

## Appendix D. Pseudo-Code for the Implementation of Fuzzification Sampling Function

function [trainingdataset,validationdataset,testingdataset,trainingdata,validationdata,testdata,knowledgeRuleBase] = fuzzyfication_sampling(totalmatrix)
read the percentage for training;
read the percentage for validation;
read the percentage for testing subsets;
read the iterations number;
calculate the number of variables;
for i = 1: iterations number
for j = 1: number of variables
[training, validation,testing ] = divide randomly the complete dataset with the chosen percentages.
end
[knowledgeRuleBase] = knowledgeRB(i,training, validation,testing,numvarinputs);
end
end

## Appendix E. Pseudo-Code for the Implementation of CrispOutputs Function

function [performance] = CrispOutputs(fis,trainingdata,validationdata,testingdata,indicesinputs,indicesoutputs,combination);
inputtrainingdata = trainingdata(:,indicesinputs(combination,:));
outputsdata = trainingdata(:,indicesoutputs(combination,:));
fisoutputstrainingdata = evaluate the performance with the inputtrainingdata variable.
calculate correlation coefficient (regression) using outputsdata and fisoutputtrainingdata;
calculate the square of the correlation coefficient (R2)
inputvalidationdata = validationdata(:,indicesinputs(combination,:));
outputsdata = validationdata(:,indicesoutputs(combination,:));
fisoutputsvalidationdata = evaluate the performance with the inputvalidationdata variable.
calculate correlation coefficient (regression) using outputsdata and fisoutputvalidationdata;
calculate the square of the correlation coefficient (R2)
inputtestingdata = testingdata(:,indicesinputs(combination,:));
outputsdata = testingdata(:,indicesoutputs(combination,:));
fisoutputstestingdata = evaluate the performance with the inputtestingdata variable.
calculate correlation coefficient (regression) using outputsdata and fisoutputtestingdata;
calculate the square of the correlation coefficient (R2);

Calculate the total performance using the complete dataset (training, validation, and testing).
Calculate correlation coefficient (regression) using totaloutputsdata and fisoutputtotaldata.
Calculate the square of the correlation coefficient (R2).

performance = [R2_total,R2_training,R2_validation,R2_testing];
end

## Appendix F. Pseudo-Code for the Implementation of InferenceEngineandDefuzzification Function

function InferenceEngineandDefuzzification(combination,trainingdata,validationdata,testdata,knowledgeRuleBase,indicesinputs,indicesoutputs)
fis = newfis(put the fis file name);
define the defuzzification method;
for i = 1: size(indicesinputs,2)
put input variable name;
assign ranges for the input variables;
for j = 1: inputs(indicesinputs(combination,i)
put name to the membership function for the input variable
put type to the membership function for the input variable
if type == ‘trapmf’
put parameters = knowledgeDataBase(j,:,indicesinputs(combination,i));
else
put parameters = knowledgeDataBase(j,[1,2,4],indicesinputs(combination,i));
end
end
end
for i = 1: size(indicesoutputs,2)
put output variable name;
assign ranges for the output variables;
for j = 1: inputs(indicesoutputs(combination,i)
put name to the membership function for the output variable
put type to the membership function for the output variable
if type == ‘trapmf’
put parameters = knowledgeDataBase(j,:,indicesoutputs(combination,i));
else
put parameters = knowledgeDataBase(j,[1,2,4],indicesoutputs(combination,i));
end
end
end
for m = 1: size(knowledgeRuleBase,1)
put antecedents;
put consequents;
put weights;
put connections;
end
namevars = concatenate(‘Vars_’,indicesinputs(combination,:));
[performances(:,:,combination)] = CrispOutputs(fis,trainingdata,validationdata,testingdata,indicesinputs,indicesoutputs,combination);
namefis = concatenate(performances(:,:,combination),’#_vars’,size(indicesinputs,2),’_’,namevars);
save the fis file: ‘*.fis’ with the namefis variable;
save the variables: ‘*.mat’ with the namefis variable;
end

## Appendix G. Pseudo-Code for the Implementation of Show Results Function

function ShowResults(numinputs,numoutputs,fisbiggerR2,subsetdata,indicesinputsdata,indicesoutputsdata)
inputsdata = subsetdata(:,indicesinputsdata);
outputsdata = subsetdata(:,indicesoutputsdata);
fisoutputsdata = evaluate the performance with the inputsdata variable.
make a confusion matrix between outputsdata and fisoutputdata;
show the matrix confusion;
make a table with the input and output variables (observed and predicted);
save the table;
end

## Appendix H. Pseudo-Code for the Implementation of Fuzzification cross Validation Function

function [trainingdataset,validationdataset,testingdataset,trainingdata,validationdata,testdata,knowledgeRuleBase] = fuzzyfication_Cross_Validation(totalmatrix,kfolds,partition_method)
CVO = cvpartition(totalmatrix(:,end-1,end);
for i = 1: CVO.NumTestSets
trIndx = CVO.training(i);
teIndx = CVO.test(i);
trainingdataset = [];
trainingdata = [];
validationdataset = [];
validationdata = [];
testingdataset = [];
testingdata = [];
for j = 1: number of variables
trainingdataset(:,:,j) = totalmatrix(trIndx,:,j);
trainingdata (:,:,j) = trainingdataset(:,1,j);
validationdataset(:,:,j) = totalmatrices(teIndx,:,j);
validationdata(:,:,j) = validationdataset(:,1,j);
end
reshape trainingdata;
reshape validationdata;
[knowledgeRuleBase] = knowledgeRB(i,trainingdata, validationdata,testingdata,numvarinputs);
end
end

## Appendix I. Pseudo-Code for the Implementation of the Main Function

function Main()
read inputs;
numinputs = sizeInputs;
read outputs;
numoutputs = sizeOutputs;
read cluster_method;
matrix = [inputs outputs];
read data partition method;
[knowledgeDataBase,ranges] = Fuzzification(matrix,cluster_method,numinputs,numoutputs);
**Case** data partition method == random sampling:
[trainingdataset,validationdataset,testingdataset,trainingdata,validationdata,testdata,knowledgeRuleBase] = fuzzyfication_sampling(totalmatrix);
**Case** data partition method == cross validation:
read partition method;
read kfolds;
[trainingdataset,validationdataset,testingdataset,trainingdata,validationdata,testdata,knowledgeRuleBase] = fuzzyfication_sampling(totalmatrix,kfolds,partition method);
for each subset
ShowResults(numinputs,numoutputs,fisbiggerR2,subsetdata,indicesinputs,indicesoutputs);
end
end

## Appendix J. Links for Downloading the Implemented Fuzzy Inference Systems for Each Dataset

https://drive.google.com/open?id=1J1i57dxTQlqJdqR--fqirG3iaNgQwW7n for Coimbra Breast Cancer Dataset (random sampling and cross validation).
https://drive.google.com/open?id=1FKOfcEmpHPyiF-OH97FIzGsgyzzKWTsV for Wisconsin Breast cancer dataset (random sampling and cross validation).
https://drive.google.com/file/d/15HBZe-20thQAgwSUVxIggQO-P6FnmNyv/view?usp=sharing for the cesarean section dataset (random sampling and cross validation).
https://drive.google.com/file/d/1jVm7fqNiSZvHh1Poi5XKzLwFRUTSaJsi/view?usp=sharing for the cryotherapy dataset (random sampling and cross validation).
https://drive.google.com/file/d/1E-BldqrIfy-8oRRX9PE35dqvVnHeboRh/view?usp=sharing for the immunotherapy dataset (random sampling and cross validation).
https://drive.google.com/file/d/1oTTVn5a5lClmh5oKm6ClI5TuVIsG73cf/view?usp=sharing for testing the implemented datasets.
